# Health-Related Quality of Life, Depression, Anxiety, and Self-Image in Acute Lymphocytic Leukemia Survivors

**DOI:** 10.4274/tjh.2015.0356

**Published:** 2016-12-01

**Authors:** Birol Baytan, Çiğdem Aşut, Arzu Çırpan Kantarcıoğlu, Melike Sezgin Evim, Adalet Meral Güneş

**Affiliations:** 1 Uludağ University Faculty of Medicine, Department of Pediatrics, Division of Pediatric Hematology, Bursa, Turkey; 2 Uludağ University Faculty of Medicine, Department of Pediatrics, Bursa, Turkey

**Keywords:** Childhood leukemia, depression, anxiety, Self-image, Health-related quality of life

## Abstract

**Objective:**

With increasing survival rates in childhood acute lymphocytic leukemia (ALL), the long-term side effects of treatment have become important. Our aim was to investigate health-related quality of life, depression, anxiety, and self-image among ALL survivors.

**Materials and Methods:**

Fifty patients diagnosed with ALL and their siblings were enrolled. The Kovacs Children’s Depression Inventory, State-Trait Anxiety Inventory, Offer Self-Image Questionnaire, and Pediatric Quality of Life InventoryTM were used for collecting data. ANOVA tests were used to determine if there were any significant differences between groups.

**Results:**

ALL survivors had higher depression, more anxiety symptoms, lower quality of life, and more negative self-image when compared to their siblings.

**Conclusion:**

Continuous diagnostic and interventional mental health services might be necessary for possible emotional side effects of treatment during and after the treatment. Rehabilitation and follow-up programs should be implemented for children during and after treatment for ALL.

## Abstract (tr)

**Amaç:**

Akut lenfoblastik lösemide (ALL) sağkalım oranlarının artmasıyla tedavinin uzun süreli yan etkileri önemli hale gelmiştir. Bu çalışmanın amacı da ALL sağkalanlarında, sağlıkla ilişkili yaşam kalitesi, depresyon, anksiyete ve kendilik imajını incelemektir.

**Gereç ve Yöntemler:**

ALL tanısı almış 50 çocuk ile onların aynı sayıdaki sağlıklı kardeşleri çalışmaya dahil edilmiştir. Verileri toplamak için, Kovaks Çocuklar için Depresyon Anketi, Durumluluk-Sürekli Kaygı Envanteri, Offer Kendilik İmajı anketi ve Pediatric Quality of Life InventoryTM kullanılmıştır. Gruplar arası farklar ANOVA yöntemi kullanılarak araştırılmıştır.

**Bulgular:**

ALL sağkalanlarının, kardeşlerine göre, depresyon ve anksiyete puanları anlamlı olarak fazladır. Ayrıca, benlik imajlarının daha olumsuz, yaşam kalitelerinin daha düşük idi.

**Sonuç:**

ALL tedavisi sırasında ve sonrasında olası duygusal yan etkiler için sürekli tanısal ve girişimsel mental sağlık servisleri gerekli olabilir. Tedavi sırasında ve sonrasında ALL’li çocuklar için rehabilitasyon ve izlem programları uygulanmalıdır.

## INTRODUCTION

Acute lymphoblastic leukemia (ALL) is the most common type of childhood cancer. Over the past decades, survival rates have improved substantially [1,2]. Among the advances in ALL treatment, Health-related quality of life (HRQL), which is a multidimensional construct that encompasses several domains such as physical, cognitive, social, and emotional functioning, was recognized as an important outcome measure of ALL survivors [3].

Bansal et al. [4] found that children with ALL have significantly poorer social, physical, and emotional health and well-being than their peers and siblings. All treatment protocols of ALL contain higher cumulative doses of asparaginase, vincristine, and corticosteroids. Significant treatment-related toxicities might develop during the treatment period. These treatment outcomes might affect HRQL adversely [5].

Besides poorer HRQL, behavioral and emotional problems, including withdrawal, depression, anxiety, and attention problems, have been reported among children with ALL [6]. Some studies determined that long-term survivors of childhood cancer experience a great number of problems with social competence and symptoms of depression compared to healthy children and siblings [7,8].

Another important area of psychological outcome that has not been studied widely is the impact of cancer on the survivor’s self-image. This has been defined as a set of self-attitudes that reflect a description and an evaluation of one’s own behavior and attributes [9]. Self-image may be influenced by a chronic illness during childhood that affects physical appearance and opportunities for social interaction [10]. Having negative self-image could be predictive of those survivors with adjustment problems [11].

In the course of intensive therapy for ALL, there is a significant impairment in quality of life in the physical and psychosocial domains, but it improves significantly after a period of time [4]. Our study includes ALL patients in remission for 2-13 years. We analyzed the time periods in 3 different groups (2-5 years, 6-10 years, and more than 10 years of survival) to determine the effect of the time after treatment on behavior and HRQL.

The aim of this study, therefore, was to investigate HRQL, self-image, depression, anxiety behaviors, and the impact of time period after treatment among ALL survivors.

## MATERIALS AND METHODS

The study group contained 50 children in the complete remission period of ALL. The control group consisted of ALL patients’ siblings. The study group (standard and medium risk group) had no history of cranial radiation. The patients were treated with the BFM-9 leukemia protocol. Intrathecal methotrexate was given for central nervous system prophylaxis. The study group was composed of 27 (54%) female and 23 (46%) male participants. The age of the groups ranged between 13 and 18 years, and the average age of the study group was 15.8±1.8 years. The average age of the control group was 14.2±0.8 years.

The control group was chosen from age- and sex-matched siblings because they shared similar social environmental and genetic features with the study group, apart from not having been diagnosed with ALL.

If the family’s monthly income was under 2000 Turkish lira (TL), participants were considered as a lower income group. If it was between 2000 and 5000, they were considered as a middle income group, and if it was above 5000 TL, they were considered as a higher income group.

The data of the study were gathered from 4 psychometrically validated self-report instruments. All of them were administered in one session to each participant separately.

The Kovacs Children’s Depression Inventory (KCDI) is filled out by the adolescent. In this 27-item scale, there are three choices for each item. The patient is asked to choose the most relevant choice for considering the last 2 weeks. Reliability and validity study of the Turkish version of the KCDI was carried out by Öy [12] and a score of 19 was identified as the cut-off level.

The State-Trait Anxiety Inventory assesses the anxiety levels of the participants. It consists of two parts. The State Anxiety Inventory (SAI) requires the individual to describe how she/he feels at a given moment and under certain conditions and to respond to the items considering her/his feelings related to that specific condition. On the other hand, the Trait Anxiety Inventory (TAI) makes individuals express how they feel in general. The total score of each scale ranges between 20 and 80. There are 4 choices for each item. High scores (more than 41 points) indicate high anxiety levels. The reliability and validity of the Turkish version of the SAI and TAI were studied by Öner and Le Compte [13].

The Offer Self-Image Questionnaire (OSIQ) was developed to identify the opinions of adolescents on self-esteem and sense of identity. Developed by Offer, Ostrov, Howard, and Dolan in 1989, the OSIQ is a 6-point Likert-type scale (choosing the answer that the individual identifies with best) and measures individuals’ adaptation in 11 different areas. The 99-item questionnaire form analyzes the self-image of adolescents in five dimensions (psychological, social, sexual, familial, and coping). Low scores (50 points and below) indicate low self-esteem. The reliability and validity of the Turkish version of the OSIQ were studied by Savaşır and Şahin [14].

The Pediatric Quality of Life Inventory (PedsQL) examines individuals’ physical, psychological, and spiritual functioning, which are the characteristics of general well-being as defined by the World Health Organization. In addition to these, the scale also emphasizes school functioning. It consists of two subscales, which are the total physical health score (TPHS) and total score of psychosocial health (TSPH), and there is a total scale score, which is the combination of these two subscales. This scale does not include a cut-off level but lower scores indicate poor quality of life. The reliability and validity of the Turkish version of the PedsQL was studied by Çakın Memik et al. [15].

This research was approved by the Uludağ University Medical Ethics Committee and therefore the research was performed in accordance with the ethical standards of the Helsinki Declaration.

SPSS 22.00 and ANOVA were used to determine if there were any significant differences between the groups.

## RESULTS

The results from patients’ and siblings’ reports are summarized in Table 1.

Mean scores of the study and the control groups for self-report instruments are shown in Table 2.

Quality of life and self-image scores of ALL survivors were lower and depression and anxiety scores were higher than in the siblings. Table 3 shows the comparison of the quality of life, depression, anxiety, and self-image scores in the groups.

There were significant differences between groups. The study group had more depression and anxiety symptoms and negative self-image. Additionally, physical, psychological, and total qualities of life were lower than in their siblings. Table 4 shows mean scores of the depression, anxiety, quality of life, and self-image of survivors in different time periods after ALL treatment. Comparison of the depression, anxiety, quality of life, and self-image scores between ALL survivors and siblings is shown in Table 5.

There were significant differences between the groups’ TvPHS, STS, TSPH, and KCDI scores according to time period after ALL treatment. Depression and quality of life scores were lower in the group of survivors 2-5 years after treatment.

## DISCUSSION

According to our study, the total quality of life score of the ALL survivors was significantly lower compared to their siblings and they had significantly lower self-concept (including the psychological, social, sexual, and familial self domains). Our study also showed that ALL survivors had significantly higher depression and anxiety symptoms than their siblings. Finally, our research revealed that the quality of life and depression scores were significantly lower among survivors 2-5 years after treatment when compared to 6-9 years and 10 years or more.

Liew et al. [16] reported that adult long-term ALL survivors had a global HRQL score similar to the general population. van Litsenburg et al. [8] reported clinically important impaired HRQL scores of ALL survivors compared to the norms. ALL treatment impairs daily activities, family life, and school success, leading to low quality of life [17]. It is known that hospitalization for chemotherapy leads to problems such as social alienation and loneliness. For a child, quality of life is likely to be compromised by the pain of the illness and treatment, lack of energy to enjoy everyday activities, and fears about the future [18]. After cancer treatment, we usually observe that children do not want to attend to school again. Parents also usually have fears about their children contracting infections in school. The idea that their children are still vulnerable might be the reason for social isolation (according to our interviews with parents, ALL survivors are rarely allowed to join social activities outside the home), which might affect children’s quality of life negatively. Self-concept findings are similar to those of other studies, such as research on self-esteem among 578 pediatric ALL survivors compared to control groups [9]. According to some other studies, adult survivors of a variety of childhood cancers were found to have significantly lower self-esteem [18,19]. However, according to Maggiolini et al. [20], long-term adolescent ALL survivors had a more positive and mature self-image compared to a healthy student group. According to our study, self-image components such as coping capacity and individual values of these children were stronger when compared to their siblings. These results indicate that patients undergoing a long and difficult treatment period, as in leukemia, may be damaged in some self-image domains, but at the same time that period may improve their capacity to cope with the problems that they encounter.

Psychological problems among cancer patients are commonly reported. Acute stress symptoms, anxiety, depression, panic attacks, and post-traumatic stress symptoms might be observed among cancer patients [21,22]. Myers et al. [5] reported that anxiety was a significant problem in a subpopulation of patients with ALL immediately after diagnosis, whereas depression remained a significant problem for at least 1 year. Kanellopoulos et al. [23] reported that levels of anxiety and depression remained significantly associated with poor quality of life. Although major psychiatric disturbances are not common among survivors of ALL, a few earlier studies showed that this population has increased risk for mental health and adjustment problems [24,25,26]. Some studies indicate that the period after treatment is characterized by a higher risk of psychosocial problems compared with the actual treatment period. Children and adolescents who were off treatment reported higher levels of depression and anxiety.

The quality of life is worse at the time of diagnosis [7]. The period after treatment is characterized by a higher risk of psychosocial problems compared with the actual treatment period. Children and adolescents who were off treatment reported higher levels of depression [27,28].

There are some limitations of this research. First of all, besides the siblings who were our control group, a randomized peer group should have also participated in this research. Meanwhile, the ALL survivors who participated in this research came from the local area. A more widespread participant group would give more information about results.

## CONCLUSION

Despite the improved survival rates, cancer still remains a potentially life-threatening condition and a major challenge for both the child and the family. During and after the course of treatment, most children experience unpleasant physical and emotional side effects. The difficulties faced by children during and after treatment affect their quality of life, social life, and emotional status negatively. Continuous diagnostic and interventional mental health services might be necessary for possible emotional side effects during and after the treatment. Rehabilitation and follow-up programs should be implemented for these children both in the course of treatment and in the long-term follow-up period.

## Ethics

Ethics Committee Approval: The study was approved by the Uludağ University Local Ethics Committee (protocol number: 2014-2/15).

## Figures and Tables

**Table 1:**
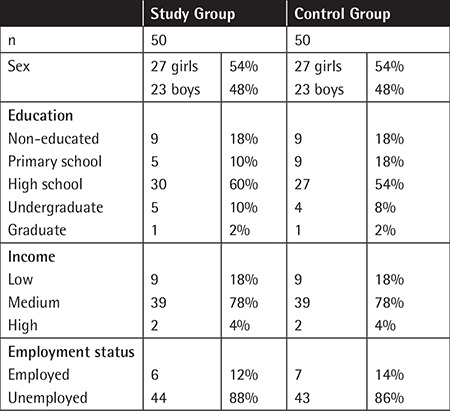
The demographic features of the study and control groups.

**Table 2:**
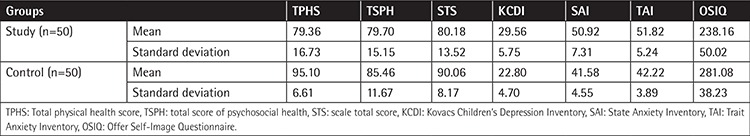
Mean scores of the study and control groups for self-report instruments.

**Table 3:**
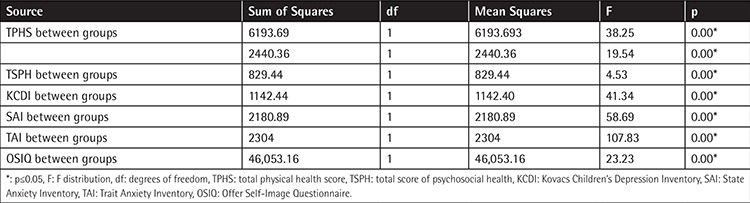
The comparison of groups’ quality of life, depression, anxiety, and self-image scores.

**Table 4:**
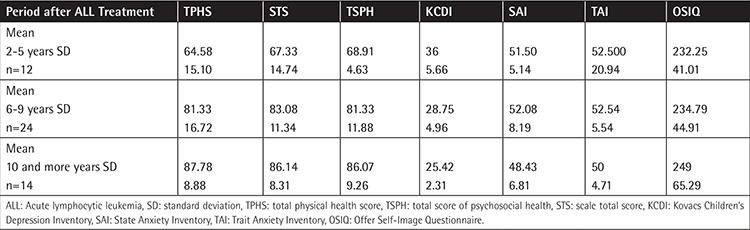
Mean scores of depression, anxiety, quality of life, and self-image regarding period after acute lymphocytic leukemia treatment.

**Table 5:**
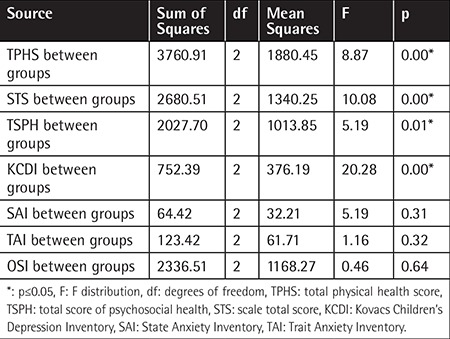
Comparison of the depression, anxiety, quality of life, and self-image scores regarding period after acute lymphocytic leukemia treatment.
